# Linking Dietary Patterns to Environmental Degradation: The Spatiotemporal Analysis of Rural Food Nitrogen Footprints in China

**DOI:** 10.3389/fnut.2021.717640

**Published:** 2021-08-30

**Authors:** Chao-Fan Xian, Cheng Gong, Fei Lu, Lu Zhang, Zhi-Yun Ouyang

**Affiliations:** State Key Laboratory of Urban and Regional Ecology, Research Center for Eco-Environmental Sciences, Chinese Academy of Sciences, Beijing, China

**Keywords:** nitrogen footprint, dietary pattern, rural food consumption, spatiotemporal analysis, China

## Abstract

**Background:** China has a large emerging economy that illustrates how dietary patterns can affect food-source nitrogen (N) cycling. The indicator of food nitrogen footprint (NF) reflects the amount of reactive nitrogen (Nr) emissions and impacts of these emissions on the environment. It is a result of food production and consumption to satisfy basic dietary demands of a given population. Different from urban food consumption with improved waste treatment, rural food consumption significantly affects the environment from food production to waste disposal. We therefore, performed a nationwide case study to link dietary patterns to environmental degradation based on rural food NF accounting.

**Methods:** The N-Calculator model was adopted to reveal the spatiotemporal characteristics of food NFs per capita, and regional food NFs related to rural diets in China from 2000 to 2019. Then, food-source Nr emissions to regional environment were quantified based on food NF accounting and relevant inventory of regional Nr emissions.

**Results:** (i) The average annual food NF per-capita in rural regions was lower than that of its national counterpart, but exhibited regional differences, mainly attributed to the dietary role of cereals. (ii) There existed significant spatiotemporal characteristics among regional food NFs that were mainly contributed by plant-derived food consumptions (73%). Sichuan, Henan, Shandong, and Hunan exhibited larger regional food NFs, and Beijing, Shanghai, and Tibet showed a growth in NFs, wherein rural diets were dominated by animal-derived food. (iii) Rural diets affected the environment by the pathways of ammonia and nitrous oxide volatilization processes, as well as Nr loss to water, accounting for a 33, 5, and 62% average of food NFs across regions. (iv) Although current rural dietary patterns suggest reliance on cereal and vegetable consumptions, more animal-derived types of food would be consumed as urbanization continues, especially in developed regions, creating a barrier for further reduction in national food NF.

**Conclusion:** The findings of this study highlight the importance of changing dietary patterns to the human health-environment dilemma. Strategies that include improvements in N recycling rates, adjustments in dietary patterns, and reductions in food wastes could mitigate regional N pollution with rural dietary shifts.

## Introduction

An essential element for biological growth, nitrogen (N) is needed by crops, animals, and humans (1, 2). Driven by population growth, the per capita consumption of plant- and animal-derived food for protein is increasing, and more N is required to support crop production (N fertilization) and livestock farming (N feeding) to meet the demand of human consumption. This has resulted in reactive nitrogen (Nr, all nitrogen species except N_2_)-related pollution through the release of Nr into the environment after N consumption, causing negative impacts on human health and the biosphere (1). Generally, the types and amounts of anthropogenic food consumption significantly affect the food production system, and their dietary patterns indirectly influence food production, as well as N requirements and losses (2). The global trend of urbanization has further changed the food demand and diet structure of citizens, and this has impacted global and regional N cycling (3). Previous research has found that the consumption of animal-derived food generally led to a higher N burden, which could be minimized through diet shift to reduce Nr and greenhouse gas (GHG) emission (4, 5). Moreover, different dietary patterns of a country or region have different environmental impacts on the environment (6).

China has a large emerging economy that illustrates how population growths, dietary shifts, and N management practices can affect long-term nitrogen use and loss from food production and consumption (5, 7). Recently, food-related research in China has boomed, involving multiple subject disciplines, with relationships between environmental degradation and public food issues (food security, food consumption, and food waste) becoming mainstream (8). However, most related studies are focused on the urban food system, with the rural food system attracting less attention, despite the fact that the latter is strongly linked to environmental degradation. With improvements of living standard in rural areas driven by national urbanization, food security is directly related to Goal 2 (no hunger) of the United Nations Sustainable Development Goals (UN SDGs), which has attracted growing attention from governments and researchers (9). Food systems in rural areas include both food production and consumption functions, which differ from those in urban areas, and are experiencing dramatic changes in terms of production patterns and dietary structure with urban-rural integration. Some studies have addressed the fact that the intensity of food-sourced Nr loss in urban areas has been higher than in rural areas (7); however, Nr reduction and recovery rates in rural areas were far lower than in urban areas. For example, rural areas in China produced nearly half of national wastewater; only 10% of this can be treated before discharge, and less Nr can be recycled from kitchen and sanitary waste, resulting in higher aquatic N load in these areas (10). Therefore, the rural environment is more vulnerable to N pollution, and the release of Nr from rural food consumption and their environmental impacts should be addressed further.

Previous studies have explored the ecological footprint, carbon footprint, and water footprint of dietary patterns (11, 12). Recently, these environmental footprints of domestic food consumption in rural China have been studied in a specific year through household-level data acquisition (9). However, relevant studies concerning the temporal and spatial characteristics of N-related environmental footprints in rural China remain lacking. One of the most important indicators reflecting the impact of human consumption on the environment (13), nitrogen footprint (NF) indicates the total amount of Nr losses to the environment from the consumption and associated production of food and energy by entities (14), and it has been widely applied to link consumers with their environmental impacts in recent case studies on different scales, namely countries (6, 15), basins (16), cities (17, 18), and institutions (19). Normally, personal N footprint can be calculated through the online platform of the N-Calculator model (www.n-print.org), in order to learn how changes in N-content resource consumption affect individual N footprints. Although the N-Calculator method sometimes cannot totally capture the entire life-cycle of N footprint, it is commonly used to calculate food NF, which occupies the majority of personal and regional N footprints (15, 20). The N-Calculator method is applied through a bottom-up approach, based on multiplying the amounts of food consumption by corresponding virtual nitrogen factors (VNFs) (6, 14). VNF is defined as N that is not contained in food products consumed by humans, but rather released into the environment during the food production process. Higher VNF means that more N applied in the food production are finally lost to the surrounding environment (21).

Diets and lifestyles have changed rapidly with urbanization during the last several decades, and dietary patterns have significantly altered anthropogenic Nr loading in the environment and tightly linked human health to environmental problems (22, 23). It is widely recognized that the diets of higher-income societies require more animal-based proteins, such as pork, beef, and dairy products (24). In China, the amount of per capita meat consumption climbed rapidly from 1970 to 2015, going from 17.4 to 55.6% (24). This increased consumption of animal-derived types of food indicates a higher personal food NF for daily diets of citizens, resulting in growth of the national food N footprint (6, 15). The above-mentioned studies revealed that food NF in urban households are higher than that in rural households; however, due to unbalanced distribution of waste recycling and treatment facilities in different regions, it is important to reduce the food NFs of rural households to improve surrounding environmental conditions and realize rural rejuvenation in China (25). Pang et al. (26) found that in Chinese agricultural watersheds, 56% of N was lost to the water environment, resulting in accelerated eutrophication, and that only 5% of N could be consumed by local residents.

However, the long-term characteristics behind rural food NFs in different Chinese regions, and the impacts of food NFs on regional environments, have not yet been satisfactorily quantified, since previous studies have mainly focused on national-scale assessments (6, 15, 20). Therefore, in this study, we adopted the N-Calculator method to determine the spatiotemporal characteristics of regional food NFs resulting from rural diets, and to quantify related-Nr losses to the environment, in order to propose plausible approaches for rural N mitigation. The specific objectives of this study are: (i) to quantify personal food NFs of rural residents and reveal food types exhibiting highest NFs in China from 2000 to 2019; (ii) to explore spatiotemporal characteristics of regional NFs across China; (iii) to analyze the impacts of rural NFs on the environment of China over the past two decades; (iv) to discuss relevant policy interventions to mitigate rural N pollution.

## Materials and Methods

### System Definition

The geographical boundary of this study is mainland China, and includes 22 provinces, five autonomous regions, and four municipalities. Hong Kong, Macau, and Taiwan regions are excluded because of data unavailability. In this study, we focused on domestic food consumption by rural residents, as it accounts for 70–80% of household food consumption (9). Changes in the environmental footprints related to eating out are not certain, so food NFs caused by eating out in rural areas were not considered in this study (9). Standard impact data (per kilogram N of NF for rural individual people, or Gg, N, and TgN for regional NF) (1 kg = 10^3^g; 1 Gg = 10^9^ g; 1 Tg = 10^12^ g) were used to calculate and discuss rural food NFs with their environmental impacts.

### Food N Footprint Calculation

Food NF represents all N losses along the chain of food production to food consumption, and the modified version of the N-Calculator model (14) was applied with respect to the rural food chain. The energy consumption of food production and consumption, and fossil fuel combustion associated with transportation and production of goods and services, were not considered in this N-Calculator approach, because they contribute a very small proportion to the total NF (6, 14), especially in the Chinese rural regions that mainly rely on conventional tillage and cooking. In this research, we chose 13 different food items, such as five plant-derived foods (cereal, legume, vegetable, fruit, and sugar), and seven animal-derived foods (pork, beef, mutton, poultry, seafood, egg, and milk). In China, edible oil is mainly produced from oil plant extraction, and it could be regarded as a plant-derived food. The total personal food NF of rural dining in China is the sum of the per capita NF of the above 13 selected foods consumed. The following equations show the method for food NF (FNF) calculation:

(1) $$\begin{array}{l} FNF=FP_{C}+FP_{P} \end{array}$$

(2) $$\begin{array}{l} FP_{C}^{j}=\sum_{i=1}^{i=n}FD_{i}^{j}\times N_{i}\times \left(1-HU_{f}\right)\times P_{j} \end{array}$$

(3) $$\begin{array}{l} FP_{P}^{j}=\sum_{i=1}^{i=n}FD_{i}^{j}\times N_{i}\times VNF_{i}\times P_{j} \end{array}$$

where $FP_{C}^{j}$ and $FP_{P}^{j}$ represent the *NFs* of food consumption and production, respectively; and *i* represents the different types of food (*n* = 13) with *N*_*i*_ as the N content of food *i*. $FD_{i}^{j}$ represent the amount of food *i* consumption by rural people per capita in the region *j*, and *P*_*j*_ presents the rural population in region *j*. *HU*_*f*_ represents the human uptake ratio of food-source N (2%) (15). *VNF*_*i*_ is the virtual N factor of food *i*, representing the amount of Nr lost to the environment per unit food-source N consumed (14), which was according to the previous research focusing on national NF estimation (15). The *VNF*_*i*_ of various foods were assumed as constants during the period of 2000–2019 (6, 15). Sewage and kitchen waste treatment with N removal technology was not considered in this study because of widespread low treatment rates across rural regions in China (6); therefore, the N consumed by human was considered to be directly released to the water environment, mainly in the form of table loss and human waste (10). The various forms of food-source Nr released to the environment can be estimated by the following:

(4) $$\begin{array}{l} E_{air}=E_{NH}+E_{NO}=FP_{P}^{j}\times P_{NH}^{j}+FP_{P}^{j}\times P_{NO}^{j} \end{array}$$

(5) $$\begin{array}{l} E_{wat}=FP_{P}^{j}\times P_{WAT}^{j}+FP_{C}^{j} \end{array}$$

where *E*_*air*_ and *E*_*wat*_, respectively, represent the parts of regional food NF loss *via* gaseous ammonia (NH_3_) and nitrous oxide (N_2_O) volatilization, and Nr loss to water, which were the main approaches for Nr entering the environment during food production and consumption (6, 27). *P*_*NH*_, *P*_*NO*_, and *P*_*WAT*_ denote the percentages of the above three N loss approaches during crop and livestock farming in the region *j* in China, which could be retrieved from the previous study that quantified regional Nr emissions with detailed emission inventory from 2004 to 2014 (28), and the data for 2004 and 2014 could be adopted to estimate *E*_*air*_ and *E*_*wat*_ for 2000–2003 and 2015–2019, because of data deficiency.

### Treatment of Missing Data

Several data were missing in raw data sources, such as the amount of various food consumption by regions in years 2013 and 2014. We adopted the method of mean value substitution to make up for missing data for these 2 years, which was previously applied to estimate the long-term Chinese household consumption of daily necessities (29), as follows:

(6) $$\begin{array}{l} D_{n}=D_{n-1}+\left(D_{n+m-1}-D_{n-1}\right)/m \end{array}$$

where *D*_*n*_ denotes missing data of food consumption in year *n, D*_*n*−1_ represents available data in year *n*−*1, D*_*n* + *m*−1_ presents available data in year *n* + *m*−*1*, with *m* presenting the number of years with missing data.

### Data Sources

Time-series data of rural food consumption per capita were reported in National Bureau of Statistics of China (30) and National Bureau of Statistics of China (31) from 2000 to 2019. Besides, relevant environmental data were collected from National Bureau of Statistics of China (32). Corresponding N content coefficients and Nr loss factors for food NF calculation were retrieved from the above-mentioned peer literature and recent survey studies (6, 15, 28).

## Results and Discussion

### Characteristics of Rural per Capita Food NFs

Table 1 shows that from 2000 to 2019, the average per capita food N consumption by rural people varied widely among food types. in particular cereal, legume, pork, poultry, seafood, and egg consumption. Food-source N intake by rural cereal consumption was decreased by 42%, but it was still the main approach for rural residents to absorb N nutrients. The consumption of vegetable presented an insignificant decreasing trend, showing a stable trend in vegetable consumption for rural diet. However, the consumption of other plant-derived food types, such as legumes and fruits, also increased, while legume consumption gradually became the main dietary N source, subsequent to cereals and vegetables. Besides, consumptions of animal-derived foods, such as pork, poultry, seafood, and egg, increased significantly. Overall, the food-source N consumed by rural residents slightly decreased from 2000 (4.61 kg N cap^−1^ year^−1^) to 2019 (4.13 kg N cap^−1^ year^−1^). According to the updated Dietary Guidelines for Chinese Residents (33), the consumption of most plant-derived foods by rural residents had met the recommended criterion of nutrition intake, but fruit consumption was far less than those required by the criterion. Animal-derived food consumptions were slightly beyond the maximum limit of the criterion, and dominated by red meat (mainly pork**)**. High-protein-food consumption, such as seafood, egg, and milk, still was not even close to the criterion.

**Table 1:** Average per capita food nitrogen (N) consumptions by rural people from 2000 to 2019, and ranges of national recommended N intake.

**Food types**	**N contents (%)**	**Virtual N factors**	**National average food N consumptions by rural people per capita (kg/yr)**					N consumptions suggested by Chinese Dietary Guidelines (kg/yr)^*^
			**2000**	**2005**	**2010**	**2015**	**2019**	**2021**
Cereal	1.36	2.1	3.31	2.77	2.41	2.04	1.93	1.24–1.99
Legume	4.67	0.95	0.27	0.15	0.13	0.43	0.57	0.43–0.60
Vegetable	0.21	7	0.24	0.21	0.20	0.19	0.19	0.23–0.38
Fruit	0.2	21	0.04	0.02	0.04	0.06	0.09	0.15–0.26
Sugar	0.37	0.77	0.01	0.01	0.01	0.01	0.01	/
Edible oil	3.11	0.87	0.22	0.19	0.20	0.31	0.30	/
Pork	2.19	3.1	0.29	0.34	0.32	0.43	0.44	0.26–0.48^**^
Beef	2.19	51.8	0.01	0.01	0.01	0.02	0.03	/
Mutton	2.22	16.5	0.01	0.02	0.02	0.02	0.02	/
Poultry	1.9	1.6	0.05	0.07	0.08	0.13	0.19	/
Seafood	1.41	4.2	0.06	0.07	0.07	0.10	0.14	0.21–0.39
Egg	1.83	0.77	0.09	0.09	0.09	0.15	0.18	0.27–0.33
Milk	0.55	5.6	0.01	0.02	0.02	0.03	0.04	0.60
Total	/	/	4.61	3.97	3.61	3.92	4.13	/

* *CNS (33)*.

** *Recommended N intake by overall livestock meat consumptions*.

**Figure 1:**
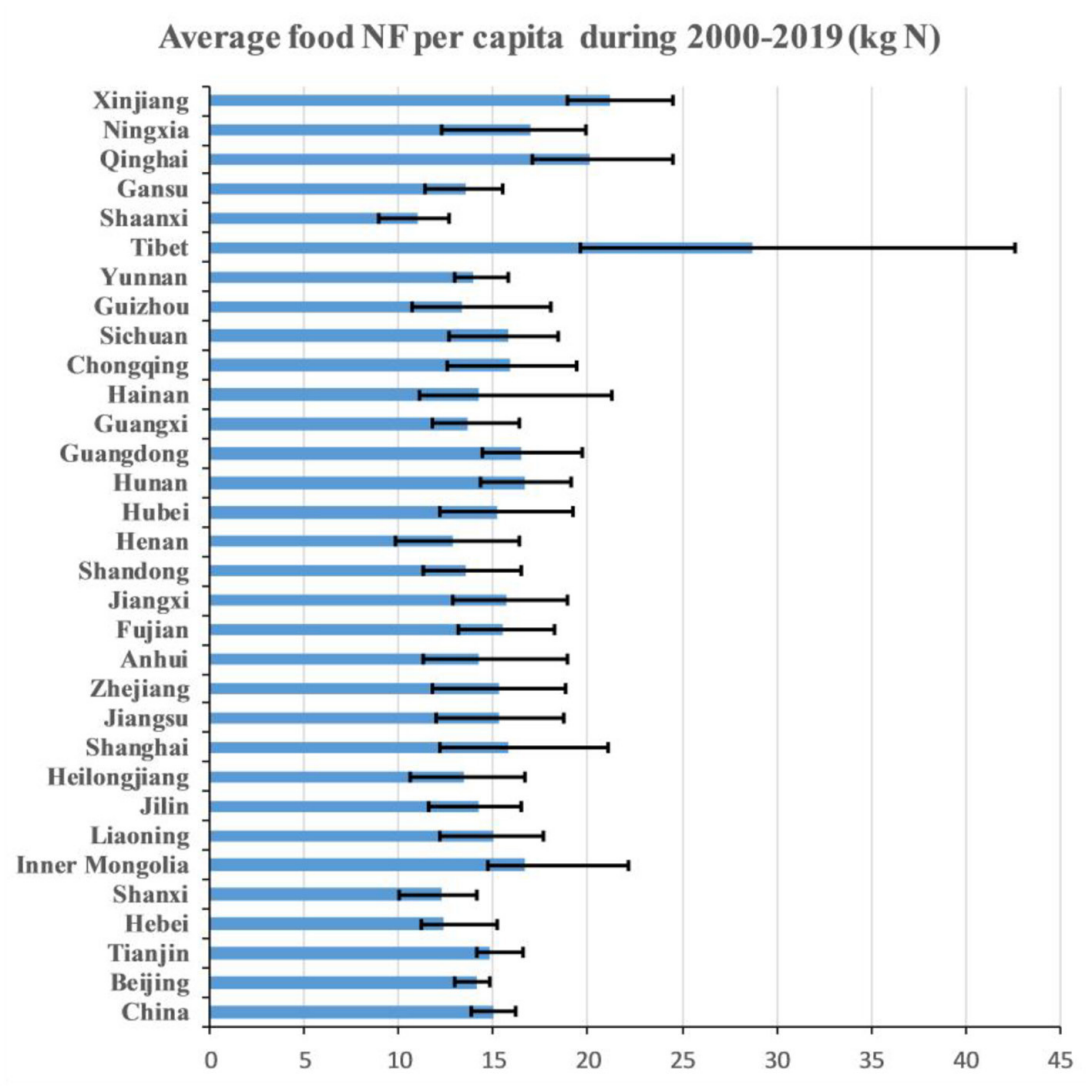
Average rural per capita food nitrogen footprints (NFs) in nation and regions with the maximum and minimum from 2000 to 2019.

Food nitrogen footprints per capita in different regions are shown in Figure 1. From the beginning of the study period (2000), the overall per capita food NF for rural residents in China was 16.47 kg N, which was lower than the national average of 21.3 kg N for both urban and rural food consumptions in the same year (6). Then, it gradually decreased to 12.94 kg N by 2012, but climbed to 16.39 kg N in 2019. The average value (15.03 kg N) for Chinese rural residents was lower than those of Austria and Germany, which were far below the Chinese average values in 2000, but still was higher than that of Tanzania.

There exited significant temporal and spatial characteristics among the rural food NFs per capita in Chinese regions (Figure 1). The average rural per capita food NFs of 77% regions fell in the range of 13–17 kg N, and extremely higher rural per capita food NFs were found in Tibet (28.75 kg N), Xinjiang (21.17 kg N), and Qinghai (20.13 kg N), where population densities are lower compared with China. Lower average rural per capita food NFs were shown in Shaanxi (10.98 kg N), Shanxi (12.21 kg N), Henan (12.83 kg N), and Hebei (13.39 kg N), which are located in North China. The “planetary boundary” per capita for food consumption is defined as a value higher than the environmental footprints induced by the food consumption of an individual, otherwise the integrity of the ecosystem of earth will be jeopardized. The planetary boundary for the nitrogen application per capita per year is around 10 kg N (34). According to the results, rural food NFs per capita in most regions from 2000 to 2019 had exceeded this “warming threshold,” except Shaanxi from 2010 to 2014. Current rural per capita diets still remain unsustainable in terms of ecological protection toward rural rejuvenation.

### Characteristics of Regional Food NFs

**Figure 2:**
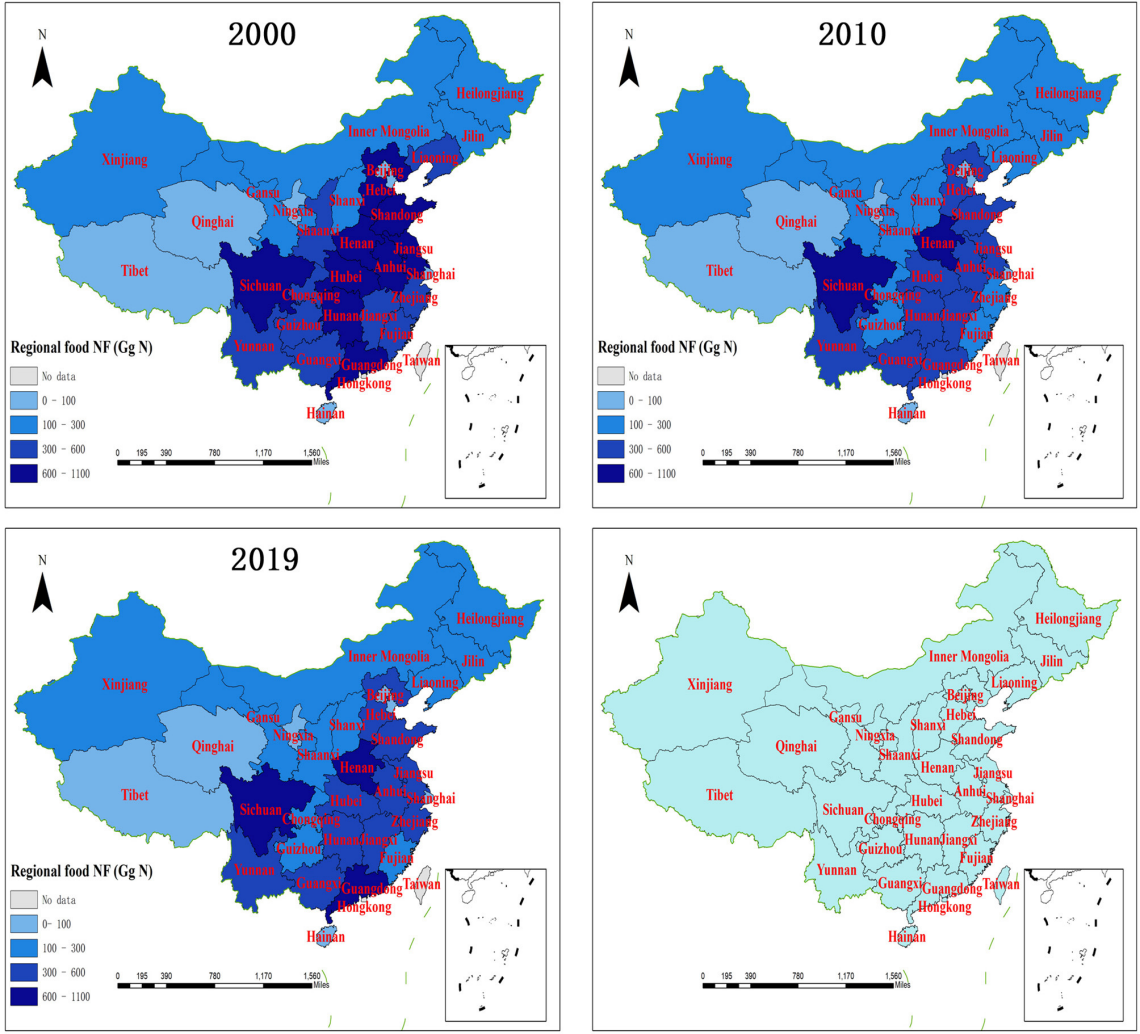
Spatial distribution of regional food NFs from 2000 to 2019.

In terms of national food nitrogen footprint contributed by rural residents, it decreased with a fluctuation from 12.95 Tg N in 2000 to 9 Tg N in 2019, with the lowest presenting in 2012 (7.77 Tg N). The regional food NFs presented significant heterogeneity across China (Table 2; Figure 2). The highest average food NF was observed in Sichuan (779.51 GgN year^−1^) from 2000 to 2019, followed by Henan, Shandong, and Hunan with average food NF higher than 600 GgN year^−1^, and the food NFs per capita of these regions were lower compared with the other regions, but their huge rural populations probably caused larger regional food NFs. On the contrary, the regions in west China with higher per capita food NFs produced less regional food NFs, such as Qinghai and Tibet, with average food NFs lower than 70 Gg year^−1^, and merely higher than those of cities such as Tianjin (39.65 GgN year^−1^), Beijing (40.44 GgN year^−1^), and Shanghai (42.16 GgN year^−1^), as well as Hainan island (61.58 GgN year^−1^). Overall, since 2000, most of the regions have reduced their rural food nitrogen footprints, with remarkable reductions in Jiangsu (52.86%), Guizhou (47.76%), Hubei (46.22%), and Jiangxi (44.06%). However, those of the above-mentioned cities, such as Shanghai, Beijing, and Tianjin, increased significantly by 75.29, 18.24, and 10.57%, respectively. Previous studies have also denoted that, the rural NF in Shanghai had increased from 2000 to 2017, attributed to the growth of food NF per capita, which would be increased by 2027 (18). Therefore, the higher personal food NF post challenges to regional NF reductions in these populous municipalities with lower percentages of rural population. As to the remote regions with the growths in both rural population and personal food NF, Tibet increased rural food NF by 30.77% due to increasing rural population and personal food NF. What should be noted is that the percentages of food production NFs lay in the range of 70 to 80% for most regions in the study period, except those for Qinghai, Tibet, Xinjiang, and Ningxia during periods 2003–2019, 2005–2019, 2012–2019, and 2014–2019. These regions had higher proportions of food production NF that exceeded 80% in these periods. Therefore, reduction in food production NF is key to mitigate regional food NFs.

**Table 2:** Average regional food nitrogen footprints (NFs) of food production and consumption in rural areas from 2000 to 2019.

**Geographical distribution**	**Regions**	**Average annual food consumption NF (Gg)**	**Average annual food production NF (Gg)**	**Average annual food NF (Gg)**	**Change in the period 2000–2019 (%)**
North China	Beijing	8.71	31.74	40.44	18.24
	Tianjin	9.72	29.93	39.65	10.57
	Hebei	134.68	358.94	493.62	−22.49
	Shanxi	63.87	159.85	223.71	−28.43
	Inner Mongolia	43.91	144.64	188.55	−13.89
North-East China	Liaoning	64.89	183.62	248.51	−37.57
	Jilin	48.43	133.26	181.69	−10.32
	Heilongjiang	61.59	163.91	225.49	−15.68
East China	Shanghai	9.75	32.42	42.16	75.29
	Jiangsu	134.40	373.93	508.34	−52.86
	Zhejiang	81.07	236.02	317.09	−17.02
	Anhui	137.79	364.42	502.20	−22.00
	Fujian	67.13	186.63	253.76	−33.07
	Jiangxi	106.20	287.87	394.06	−44.06
	Shandong	175.20	469.87	645.06	−38.03
Central and Southern China	Henan	204.23	558.97	763.20	−39.42
	Hubei	119.70	329.97	449.67	−46.22
	Hunan	165.66	459.16	624.83	−37.42
	Guangdong	152.49	425.70	578.19	−5.44
	Guangxi	107.17	286.50	393.67	−16.39
	Hainan	16.13	45.46	61.58	−11.23
Southwest China	Chongqing	60.60	161.54	222.14	−37.15
	Sichuan	209.28	570.24	779.51	−31.34
	Guizhou	87.62	236.67	324.29	−47.76
	Yunnan	109.01	299.55	408.56	−32.57
	Tibet	10.96	54.82	65.78	30.77
Northwest China	Shaanxi	64.25	160.48	224.72	−37.15
	Gansu	61.74	161.07	222.81	−27.19
	Qinghai	10.97	51.66	62.63	−21.79
	Ningxia	18.62	49.36	67.98	−14.87
	Xinjiang	50.16	215.36	265.52	1.06

**Figure 3:**
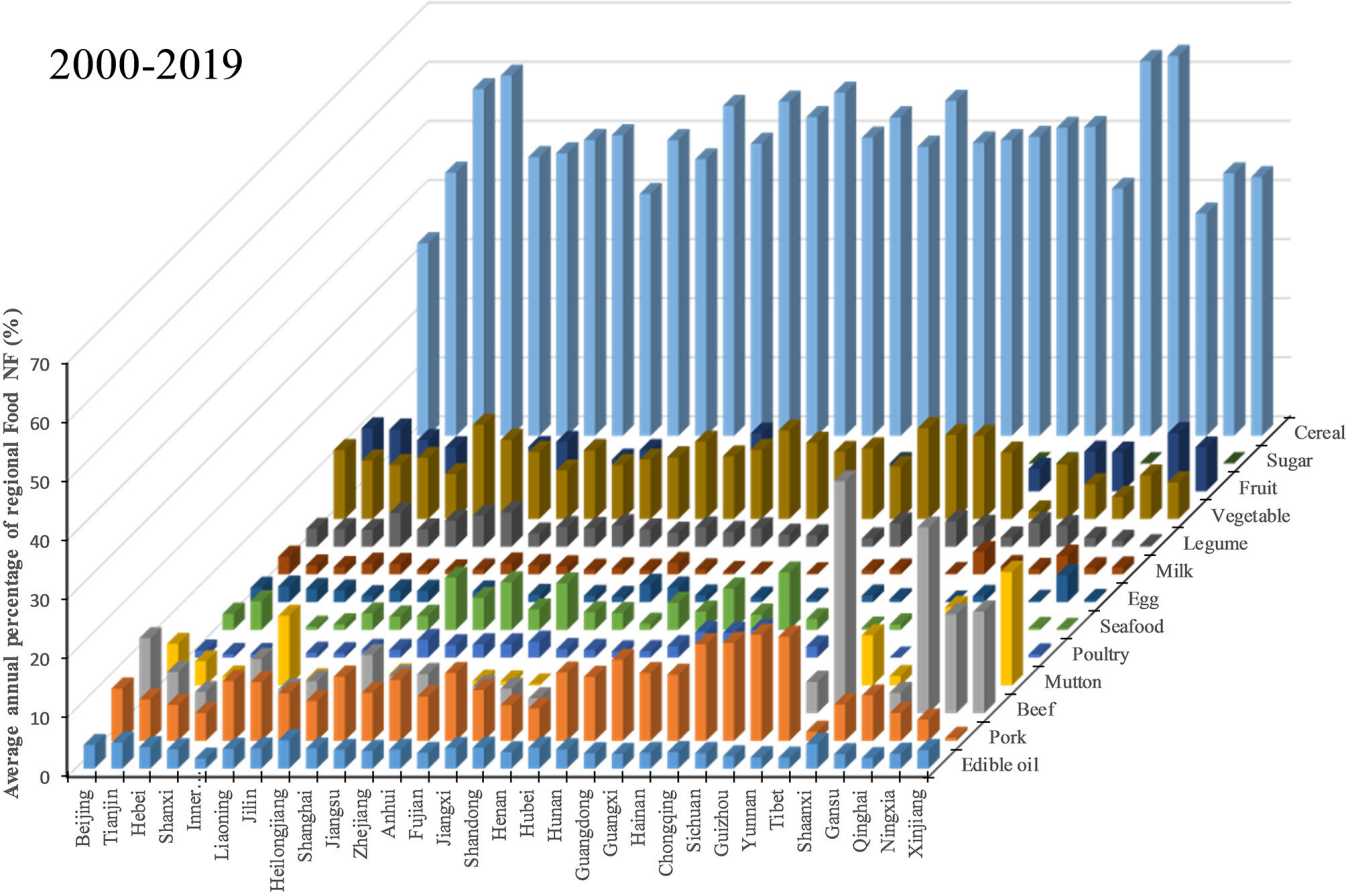
Average annual percentage of regional food NFs attributed to various food types from 2000 to 2019.

It is undeniable that cereal consumption during rural dining contributed most to regional food nitrogen footprints (Figure 3). From 2000 to 2019, rural cereal consumption contributed 33–64% of regional food NFs, with an average of 50%, indicating that it was still the main source of rural Nr release. However, food NFs of some of the regions did not rely heavily on cereal consumption, such as Beijing and Qinghai, with percentages lower than 40%. Apart from cereal, consumptions of plant-derived types of food, such as vegetables and fruits, occupied relative higher proportions, ranging from 1 to 16% (average 10%) and 1 to 11% (average 6%), respectively, which exhibited significant regional differences. Regional food NFs caused by vegetable consumptions were prominent in Liaoning (16%) and Chongqing (15%) municipalities, but were shrinking in Tibet (1%) and Qinghai (4%). Fruit consumptions contributed more to regional food NFs in Beijing (11%), Tianjin (10%), Ningxia (10%), Gansu (7%), and Xinjiang (7%), and even exceeded the percentage of local vegetable consumptions. Legume (average 3%) consumption was higher in Shanxi (6%), Heilongjiang, (6%) and Jilin (5%), and was lower in Xinjiang (.20%), Ningxia (1%), Qinghai (1%), and Hainan (1%) in terms of regional NF production. Sugar consumption presented lower (average 0.1%) across China; however, the proportion of edible oil (average 3%) contributing to regional NFs was equal to that of legume, and higher contributions of edible oil were witnessed in Heilongjiang (5%), Tianjin (4%), Beijing (4%), Shaanxi (4%), and Hebei (4%), which are mainly located in the North China Plain. The overall contribution of plant-derived types of food for rural diet to regional NFs was around 73% from 2000 to 2019.

Pork and beef were the main animal-derived food causing regional nitrogen footprints, and they occupied average percentages of 9% and 8% of regional food NFs across the period (Figure 3). The percentage of pork-source NFs were higher in most regions in Southwest China, such as Guizhou (18%), Yunnan (18%), Sichuan (17%), and Chongqing (16%), and extremely lower in Xinjiang (1%) and Tibet (2%), animal-derived food NFs, which were mainly attributed to beef consumption with percentages of 39 and 17%, respectively. Apart from Xinjiang and Tibet, beef also played important roles in regional food NFs of Qinghai (31%), Ningxia (17%), and Beijing (13%). Hotspots of regional food NFs contributed by mutton were found mainly in Xinjiang (19%), Qinghai (13%), Inner Mongolia (12%), Tibet (8%), and Beijing (7%), but mutton consumption merely contributed an average of 3% to overall regional food NFs, similar to the consumptions of edible oil and legume. “White meat” consumption by rural residents contributed less to regional food NFs, such as poultry (average 2%) and seafood (average 3%). The hotspots of poultry contributing to regional food NFs were located in South China, such as Hainan (5%), Guangdong (4%), and Guangxi (4%). In terms of seafood consumption, coast regions in Southeast China were the hotspots, such as Hainan (10%), Shanghai (9%), Zhejiang (8%), Fujian (8%), and Guangdong (7%), while inland regions consumed less seafood for their rural diet. High-protein foods, such as egg (average 1%) and milk (average 1%), play frail roles in rural food NFs. What should be noted is that the contribution of animal-derived types of food to regional NF was higher than that of cereal in some of the regions, such as Qinghai, Tibet, and Beijing, which means that animal-derived types of food contributed most to food NFs in these regions. In addition, animal-derived types of food contributed (around 40%) equally as cereal to the rural food NF in Shanghai. The cases in Beijing and Shanghai implied the challenges to reduce regional NF in more affluent and urbanized regions with rural dietary change toward more animal-derived food consumption.

Previous studies have found that in terms of overall food production in China, Henan, Shandong, and Heilongjiang were the crucial provinces for the mitigation of food-related environment pollution at the national level, concerning improved policies for the production of livestock, vegetables, fruits, and cereals (35). In this study, we focused on food NFs resulting from rural dining, and addressed that Sichuan, Henan, Shandong, and Hunan were the key provinces for the mitigation of food-related N pollution, in terms of cereal and pork consumptions. The results could be supplements to previous studies from the perspective of rural diet change.

### Fate of Regional Food NFs

Recent studies have shown that from 1990 to 2012, Nr losses to the atmosphere increased in rural areas, mainly in the form of NH_3_ and N_2_O volatilization resulting from crop and livestock farming, and that the increase in reactive nitrogen losses to hypersphere was dominated by surface runoff, leaching, and erosion in croplands, as well as nitrogen-rich waste discharge from livestock breeding (7, 27, 36). Therefore, we evaluated the impacts of regional food NFs on the environment by quantifying various types of N release induced by rural diets, based on the results of the previous study (28).

In view of the food nitrogen footprints of 31 regions, NH_3_ volatilization and Nr loss to water were the main approaches for rural diets to affect regional environments. From a perspective of regional average, Nr loss to water occupied more than 60% of regional food NFs, but it had decreased from 63 to 61% by 2019, and the overall proportion of NH_3_ volatilization remained relatively stable (average 33%) throughout the period. The percentage of NF *via* N_2_O volatilization was lower, but it gradually climbed from 4 to 5%, attributed to denitrification from strengthened fertilization in 68% of the regions, especially in regions with intensive crop farming (Anhui, Jiangsu, Heilongjiang, and Hunan). This approach of N_2_O leakage creates a barrier for national effort to mitigate climate change, since the global warming potential (GWP) of N_2_O is much higher than that of carbon dioxide (CO_2_) (1, 5).

**Figure 4:**
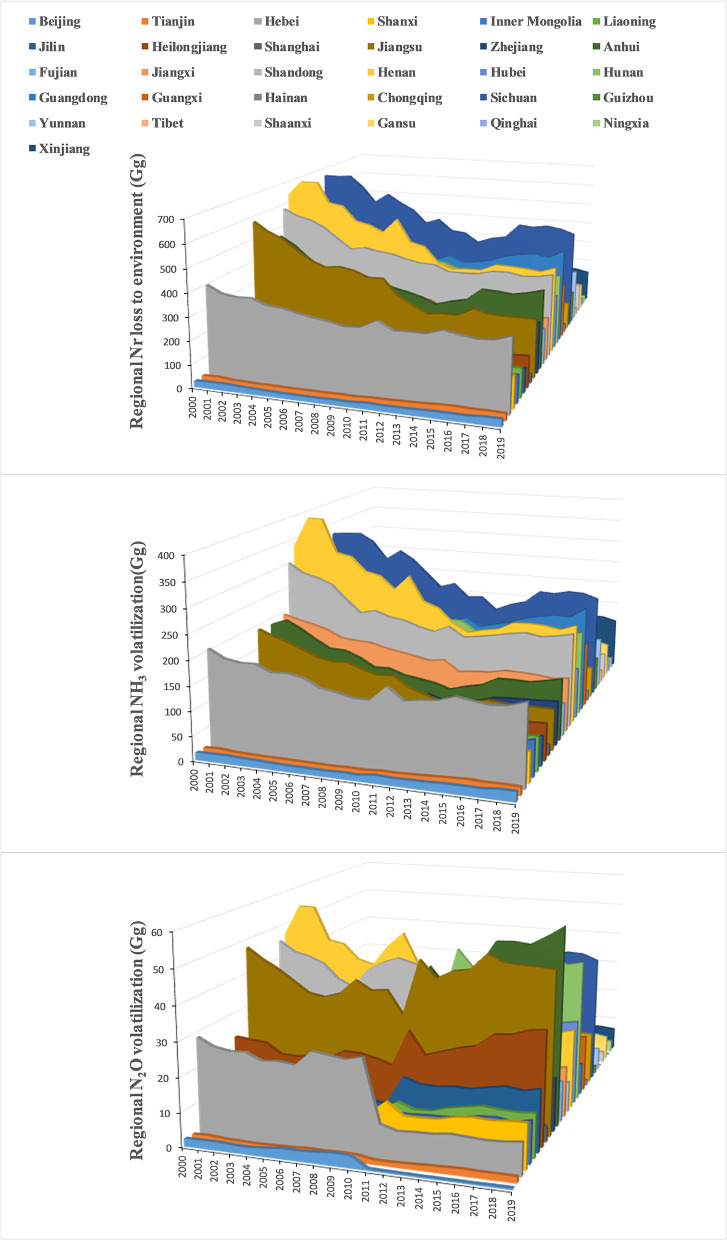
Dynamics in the various Nr loss to environment resulting from regional food NFs related to rural diet from 2000 to 2019.

Considering regional characteristics in the fate of rural food NFs (Figure 4), Sichuan (average 503 Gg N), Henan (average 475 Gg N), Shandong (average 411 Gg N), Hunan (average 380 Gg N), Guangdong (average 369 Gg N), Jiangsu (average 350 Gg N), Anhui (average 340 Gg N), and Hebei (average 315 Gg N) were the hotspots of diet-related Nr loss to water. In 2019, reductions in aquatic Nr losses were significant in Jiangsu (56%), Hubei (48%), Guizhou (46%), and Jiangxi (45%), while growth in Nr loss could be found in affluent municipalities such as Shanghai (61%), Beijing (19%), and Tianjin (9%), and the remote region Tibet (21%). Overall, rural Nr loss to water declined by 32% nationwide, from 8,304 to 5,651 Gg N, but it still posed a greater threat to regional water environment than that induced by urban diet, since less waste water was collected and treated in rural dwellings for people dining. A recent study supposed that in 2012 only 6% of rural sewage could be treated compared with urban areas (75%) (7), which underlined significant differences in food consumption NF resulting from rural and urban diets.

Reactive nitrogen loss via NH_3_ volatilization was different across regions. During the study period, rural residents in Henan contributed most to food-source reactive nitrogen (average 253 Gg N) in the form of NH_3_, followed by those in Sichuan (average 245 Gg N), Hunan (average 208 Gg N), Guangdong (average 191 Gg N), and Hebei (average 161 Gg N). Remarkably, Xinjiang increased its NH_3_ volatilization level significantly with higher aquatic Nr loss. Meanwhile, Jiangsu (average 43 Gg N), Hunan (average 36 Gg N), Henan (average 35 Gg N), Anhui (average 35 Gg N), and Sichuan (average 32 Gg N) produced more gaseous Nr though N_2_O volatilization, and 42% of the regions reduced this Nr releasing during the study period, with remarkable reductions in Beijing, Hebei, Shandong, and Shaanxi.

### Strategies for Rural Food N Footprint Reduction

**Figure 5:**
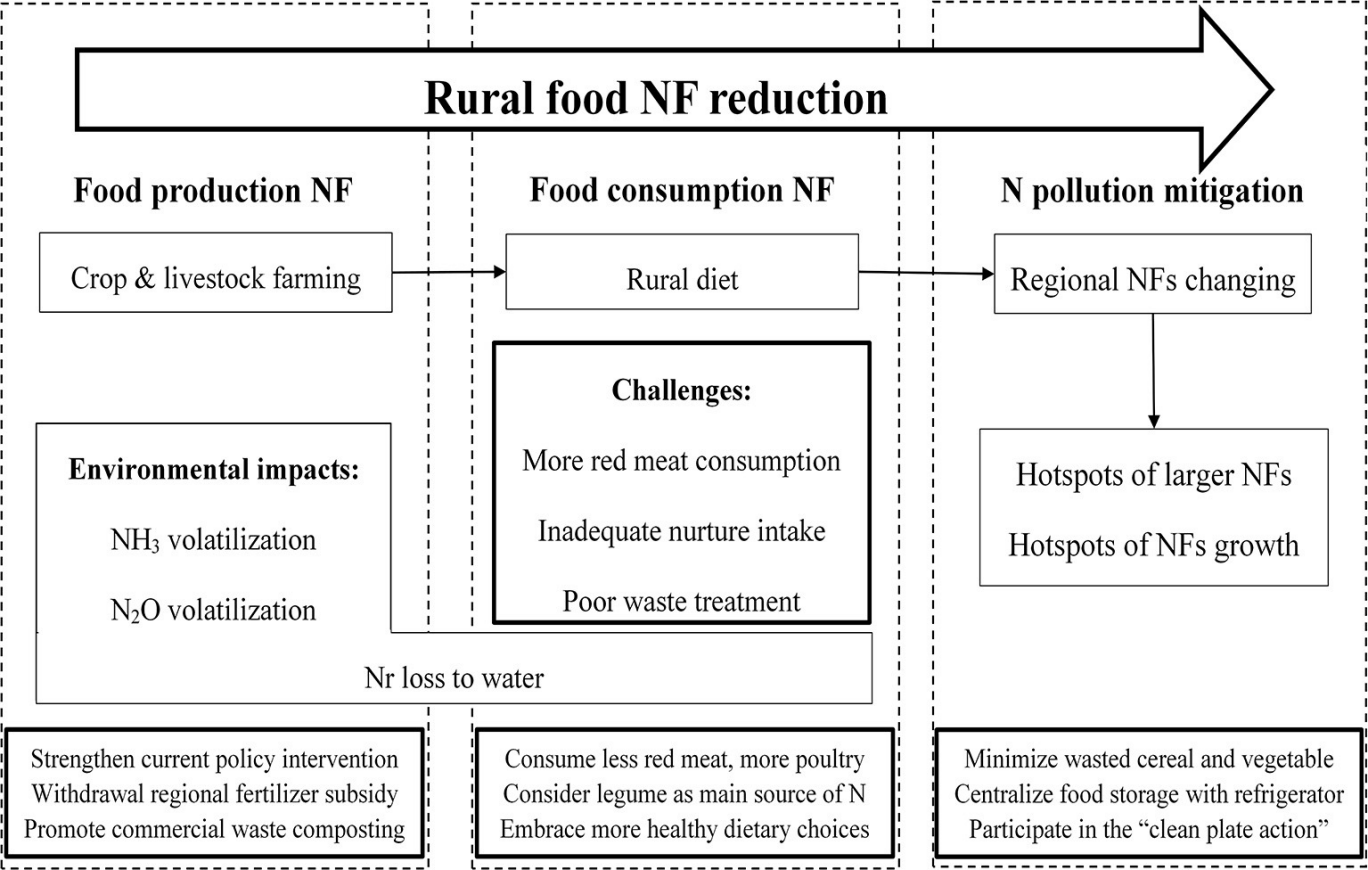
Flowchart showing the mechanism for reducing food NF addressed by relevant strategies.

With continued urbanization, potential increase in food nitrogen footprint of rural areas driven by more animal-derived food consumption or strengthened fertilization creates a challenge to the mitigation of regional N pollution. This calls for improvement in food-source N management toward more integrated approaches at the regional scale across the country. To link the dietary pattern shift to environmental degradation concerning rural food NFs (Figure 5), several strategies for food NF reduction at different stages should be addressed as follows:

#### Nitrogen Recycling Improvement

According to the results, the national food NF contributed by rural residents declined from 2000 to 2012, and then remained relatively stable in 2019. It showed that further reduction in rural food NF failed to be achieved in the past decade. In China, the overall N use efficiency is very low in crop and animal food-chain from production to consumption, with over 80% of N lost to the environment, because of higher N chemical fertilization and poor manure management. Thus, the VNFs of food production in China were generally higher than those of other countries (6, 34). Improving N recycling rate could be an economical and effective approach to recycle more “escape” N from food production to reduce extra N inputs for crop farming, minimizing VNFs. However, the food-source N recycling rate was widely lower across rural areas, and the recycled rate of the national food system decreased from 20 to 16% from 1990 to 2009 (15). This shrink in wasted N recycling probability could not be mitigated in the following years without improved technology support and stricter policy interventions.

In this sense, to tackle rural nitrogen pollution, various nitrogen-oriented regulations need to be developed in different regions. According to the results, cereal production was the main source of rural food NFs across regions, and it was supposed to have a large potential for reducing N fertilization than P fertilization (35). However, current agriculture in China still relies on synthetic fertilization, and there exist wide differences in the degree of intensified agriculture among regions. The heavier fertilization (paddy farming) in Southern and Central China, such as in Sichuan, Henan, Hunan, Guangdong, Jiangsu and Anhui, has led to significant N surplus, resulting in more Nr loss to air and water. In these regions, farmers prefer frequent fertilization that is subsidized by the government. Meanwhile, less economic returns, technical restriction, and lack of awareness may be the main factors driving the low utilization rate of recycled organic fertilization in rural areas. Moreover, with urbanization, the rural diet will continue to gradually shift to animal-derived foods with improvements in rural living standard, which has significantly accelerated the demand for animal feed, resulting in surplus in Nr budget due to the current poor management of animal wastes (7). Without proper collection and treatment, direct discharge of animal manure to rivers is the main sources of aquatic Nr release, since intensive animal production systems commonly are widely separated from crop production systems (27), causing geographical obstacles to manure recycling in farmlands.

Therefore, recycling of manure waste should be promoted by relevant policy interventions, and reasonable financial aids. The launch of “Livestock and Poultry Manure Utilization Action Program (2017–2020)” in late 2017, which aimed to improve manure recycling to minimize the environmental pollution resulting from livestock farming (27), should be strengthened in rural areas with heavier fertilization. Besides, fertilizer subsidies could gradually be substituted by more incentive funds from local governments for recycling animal wastes in farms. Pork farming is still the main intensive breeding system in plain areas, and relevant facilities for captive breeding and commercial composting with gas collection vessels could be built nearby farmlands, providing treated organic N for crop farming through centralized collection of livestock and human excrements (28). These facilities could eliminate geographical obstacles for manure recycling in farmlands, and could be widely built in plain areas in Sichuan, Henan, Hunan, Shandong, and Guangdong, areas with higher food NF production. The promotion of organic N recycling in rural regions could contribute to zero growth of chemical fertilizer use in China (7).

#### Dietary Adjustment

Since 1982, the consumption of animal-derived foods by both urban and rural residents has dramatically increased. In 2012, the percentages of seafood and poultry consumption by rural residents were still low, resulting in inadequate nutrient intakes of vitamin A, calcium, and fatty acids (*n*-3) (33). With the urban-rural gap gradually shrinking in terms of income level during urbanization, the demand for more nutrient consumption by rural people was expected to meet the recommended standard of Dietary Guidelines for Chinese Residents (33), which would stimulate rural diets to become richer in animal products, similar to those of urban residents (16). However, in recent years, individual food consumptions in urban areas have shifted toward more healthy diets attributed to the increasing health awareness of citizens (7). The lack of this awareness among rural residents may result in constantly pursuing a higher share of animal-food in their dietary compositions, which brought about challenges to rural NF reductions by means of dietary adjustments.

It should be pointed out that diet adjustments for more nutrient intakes are not limited to increasing meat consumption but also include slight shifts in the composition, quality and sources of foods. Based on the results of this study, guiding dietary shift to lower consumption of animal-derived food, and substituting “red meat” with low-N “white meat” (poultry or seafood), have been proposed as suitable solutions for the current rural diet in China. Given that seafood is not a popular rural diet in the inland regions, it is proper to prioritize poultry consumption, since it provides similar protein content as pork but produces less NF, being an N-efficient animal food choice with lower VNF (1.6) compared with pork (3.1), mutton, (16.5), and beef (51.8). Legumes, vegetables, fruits and eggs have lower food NFs, so their intakes for rural daily diet could be increased. Given that the economic costs for egg are higher, and that storage periods for vegetable and fruit are short, legumes could be a more protein-efficient choice and become the main dietary N source for rural resident next to cereal, especially in the northwest region that includes Xinjiang, Ningxia, and Qinghai. Cooperating with less “red meat” consumption, shifts in rural dietary patterns will significantly minimize Nr loss, especially for younger people who consume more meat (22), corresponding to “what a person chooses to eat does make a difference” (37).

As to the rural diet in more affluent regions that are dominated by animal-food consumption, such as Beijing, Shanghai and Tianjin, substituting egg and milk for “red meat” not only contributes to the reduction in food NFs, but also reduces obesity, since there exists a positive association between “red meat” consumption and obesity, especially in China, with rapid increase in the number of overweight and obese adults (37, 38). In terms of rural diet in remote and less-developed regions, it is the best way to encourage rural people to produce and consume poultry, eggs and legumes with less VNFs. However, in general situations, people who live below or near the poverty level do not have this luxury of choice because of poverty and ignorance. The national promotion of a “poverty alleviation policy,” to a certain extent, can alleviate food-source Nr loss to environment in rural areas. Overall, dietary adjustment toward more feasible and healthy diets could substantially reduce Nr losses, as well as improve public health.

#### Food Waste Reduction

Food waste reduction is stated as one of the most important goals to promote eco-civilization and sustainable development in China (25). Recently, some studies found that rural households generated more food wastes than their urban counterparts based on provincial surveys, and that plant-based food wastes, half of which were attributed to vegetables, were the main components of rural household waste generations (25, 39, 40). Meanwhile, wasted cereal was regarded as the food type with the most embedded environmental impacts, including land, water, and carbon, nitrogen, phosphorus footprints (34, 39). Therefore, wasted cereal and vegetables are important contributors to N pollution, since they currently are the main food types for rural diet. In terms of plant-derived food consumption, increasing legume (lower VNF) with storage stability for rural diet could reduce the proportion of cereal and vegetation consumptions, since large amounts of vegetable are probably lost because of inadequate storage and refrigeration facilities in rural areas. However, some studies showed that increasing the intake of vegetables and fruits rich in essential micronutrients is needed to achieve sustainable diets for human nutritional requirements, but their consumptions were lower globally (38). In China, the consumption of them is still lower than the criterion of Dietary Guidelines for Chinese Residents (33), so the trends in rural dietary shifting toward national nutrition intake standards would prompt increases in the consumption of vegetables and fruits in the future.

Accordingly, corresponding countermeasures concerning residential behavior, storage facility, and policy intervention, which should be promoted by regional governments, were put forward as follows: First, moderate purchase of vegetables and fruits enough for a daily diet should be encouraged. The relatively lower cost of vegetables and fruits with big discounts always resulted in excess purchasing, causing higher waste generation, especially for low-income households (25). Second, rural households in residential communities (or villages) could be equipped with larger refrigeration facilities for centralized food storage. Household refrigerators play a key role in food preservation, especially for vegetables and fruits, but the storage space of a household refrigerator is limited, and less rural households can afford a commercial refrigerator with greater storage capacity. Thus, the relevant refrigerating facilities could be supported by local governments and then rented to rural households, minimizing pre-consumption wastes. Last but not least, participating in the “clean plate action” is an effective approach for rural residents to reduce Nr loss during food production and consumption, corresponding to the recent launch of “anti-food waste law” in April 2021 in China, which provides a long-term legal system of better rules on daily catering to reduce food waste toward national food safeguard. Young people should be the target of propagandizing for “clean plate action,” since they commonly generate more food wastes (25). Overall, food waste reduction, especially for wasted vegetables, could be an effective approach to reduce food NFs in rural areas.

## Concluding Remarks

Dietary patterns are proposed as important drivers of environment degradation, as even the Mediterranean Diet, which is widely recognized as a healthy dietary pattern, still has negative impacts on the environment (11). In China, rural dietary patterns included less animal-derived foods with higher VNFs compared with urban diets, but a shift in their dietary patterns in the direction of meat-based diet, would inevitably cause a more serious N pollution. With development of urban-rural integration, the differences between the diets of urban and rural residents may be eliminated gradually, and rural diets are shifting to include more livestock products along with remaining stable consumption of cereals and vegetables, resulting in higher food NFs that are mainly released to the environment. In the future, this situation would be more popular, especially with the catering of young people in developed regions, creating a barrier for further reduction in the national food NF.

This case study, based on the N-Calculator method concerning rural food consumption, provided a holistic view of the spatial-temporal characteristics of regional food NFs related to rural dietary patterns. Similar to previous studies, several inevitable limitations may deliver uncertainties to food NF calculation, such as lack of first-hand food survey data and local VNFs for different regional NF accounting. Current food NF calculation in this study was mainly based on the official statistical data, which are acknowledged as the best available data for quantifying N remissions in China, with an error rate of ±5% (5). Besides, the ignorance of infrastructures related to waste treatments, which were gradually promoted in the rural areas along with prominent urban-rural integrations, may cause biased results. These could be overcome by more case analyses for specific regions in the future. However, these uncertainties would not significantly affect the spatiotemporal analysis of regional food NFs, because uniform parameters were adopted throughout the calculation process. In terms of novelty, this study provided a first attempt to link dietary patterns to N pollution induced by regional food NFs, aiming at elucidating the impacts of dietary patterns on environmental degradation.

We concluded that with population urbanization, the rural population in China presented declined, but the trend in rural food NF decrease was not significant especially after 2012. Despite the overall rural food NF per capita being not higher compared with its national counterpart, it still remained unsustainable across regions, and the shift in rural dietary patterns plays a key role in reducing regional food NFs. Cereal consumption is still the dominant source of rural NFs, and the overall contribution of plant-derived foods to regional NFs is around 73%. Sichuan, Henan, Shandong, Hunan, and Guangdong exhibited larger regional NFs, and the increase in regional NFs were merely presented in Beijing, Shanghai, and Tibet, the rural diets of which were dominated by animal-derived foods. Regionally, NH_3_ and N_2_O volatilization, as well as Nr loss to water, respectively accounted for average 33, 5, and 62% induced by rural food NFs. Strategies that include improvement in N recycling rate, adjustment in dietary patterns, and reduction in food waste could mitigate regional N pollution, with a shift in dietary preferences. Based on rural food NF accounting, this study highlights the view that dietary patterns have impacts on the human health-environment dilemma, which is important for sustainable rural rejuvenation with urbanization in China.

## Data Availability Statement

The raw data supporting the conclusions of this article will be made available by the authors, without undue reservation.

## Author Contributions

C-FX and Z-YO conceived the study. C-FX, CG, and FL collected the data. C-FX and CG analyzed the data. C-FX and LZ visualized the data. Z-YO supervised and reviewed the manuscript. All authors contributed to the article and approved the final vision.

## Conflict of Interest

The authors declare that the research was conducted in the absence of any commercial or financial relationships that could be construed as a potential conflict of interest.

## Publisher's Note

All claims expressed in this article are solely those of the authors and do not necessarily represent those of their affiliated organizations, or those of the publisher, the editors and the reviewers. Any product that may be evaluated in this article, or claim that may be made by its manufacturer, is not guaranteed or endorsed by the publisher.
